# Predictors of fatality in pandemic influenza A (H1N1) virus infection among adults

**DOI:** 10.1186/1471-2334-14-317

**Published:** 2014-06-10

**Authors:** Önder Ergönül, Servet Alan, Öznur Ak, Fatman Sargın, Arzu Kantürk, Alper Gündüz, Derya Engin, Oral Öncül, Ilker Inanc Balkan, Bahadir Ceylan, Nur Benzonana, Saadet Yazıcı, Funda Şimşek, Nuray Uzun, Asuman İnan, Eren Gulhan, Meral Ciblak, Kenan Midilli, Mustafa Ozyurt, Selim Badur, Serap Gencer, Ozcan Nazlıcan, Serdar Özer, Nail Özgüneş, Taner Yıldırmak, Turan Aslan, Pasa Göktaş, Nese Saltoğlu, Muzaffer Fincancı, Ali Ihsan Dokucu, Haluk Eraksoy

**Affiliations:** 1 Department of Infectious Diseases and Clinical Microbiology, Koç University, School of Medicine, Istanbul, Turkey; 2 Department of Infectious Diseases and Clinical Microbiology, Ministry of Health, Haseki Training and Research Hospital, Istanbul, Turkey; 3 Department of Infectious Diseases and Clinical Microbiology, Ministry of Health, Kartal Training and Research Hospital, Istanbul, Turkey; 4 Department of Infectious Diseases and Clinical Microbiology, Ministry of Health, Göztepe Training and Research Hospital, Istanbul, Turkey; 5 Department of Infectious Diseases and Clinical Microbiology, Ministry of Health, Okmeydanı Training and Research Hospital, Istanbul, Turkey; 6 Department of Infectious Diseases and Clinical Microbiology, Ministry of Health, Şişli Etfal Training and Research Hospital, Istanbul, Turkey; 7 Department of Infectious Diseases and Clinical Microbiology, Ministry of Health, Haydarpaşa Training and Research Hospital, Istanbul, Turkey; 8 Department of Infectious Diseases and Clinical Microbiology, Gülhane Military Medical Academy, Haydarpaşa Training Hospital, Istanbul, Turkey; 9 Department of Infectious Diseases and Clinical Microbiology, Istanbul University, Cerrahpaşa Medical Faculty, Istanbul, Turkey; 10 Department of Infectious Diseases and Clinical Microbiology, Ministry of Health, Istanbul Training and Research Hospital, Istanbul, Turkey; 11 Department of Microbiology and Clinical Microbiology, National Influenza Reference Laboratory, Istanbul University, Istanbul Faculty of Medicine, Istanbul, Turkey; 12 Microbiology Department, Istanbul University, Cerrahpaşa Medical Faculty, Istanbul, Turkey; 13 Clinical Microbiology Department, Gülhane Military Medical Academy, Haydarpaşa Training Hospital, Istanbul, Turkey; 14 Ministry of Health, Istanbul Health Directorate, Istanbul, Turkey; 15 Infectious Diseases and Clinical Microbiology Department, Bezmialem Vakif University, Faculty of Medicine, Istanbul, Turkey; 16 Infectious Diseases and Clinical Microbiology Department, Istanbul University, Istanbul Faculty of Medicine, Istanbul, Turkey

## Abstract

**Background:**

The fatality attributed to pandemic influenza A H1N1 was not clear in the literature. We described the predictors for fatality related to pandemic influenza A H1N1 infection among hospitalized adult patients.

**Methods:**

This is a multicenter study performed during the pandemic influenza A H1N1 [A(H1N1)pdm09] outbreak which occurred in 2009 and 2010. Analysis was performed among laboratory confirmed patients. Multivariate analysis was performed for the predictors of fatality.

**Results:**

In the second wave of the pandemic, 848 adult patients were hospitalized because of suspected influenza, 45 out of 848 (5.3%) died, with 75% of fatalities occurring within the first 2 weeks of hospitalization. Among the 241 laboratory confirmed A(H1N1)pdm09 patients, the case fatality rate was 9%. In a multivariate logistic regression model that was performed for the fatalities within 14 days after admission, early use of neuraminidase inhibitors was found to be protective (Odds ratio: 0.17, confidence interval: 0.03-0.77, p = 0.022), nosocomial infections (OR: 5.7, CI: 1.84-18, p = 0.013), presence of malignant disease (OR: 3.8, CI: 0.66-22.01, p = 0.133) significantly increased the likelihood of fatality.

**Conclusions:**

Early detection of the infection, allowing opportunity for the early use of neuraminidase inhibitors, was found to be important for prevention of fatality. Nosocomial bacterial infections and underlying malignant diseases increased the rate of fatality.

## Background

In April 2009 a novel strain of human influenza A of swine origin, identified as A(H1N1)pdm09 virus, rapidly spread worldwide, and in early June 2009 the World Health Organization (WHO) raised the pandemic alert level to phase 6 [1]. Many northern countries experienced the first wave of outbreak during late spring and summer months, followed by an early 2009 fall influenza season [2]. The first laboratory confirmed case in Istanbul was reported in May 2009 [3]. According to the Ministry of Health of Turkey, approximately 6.5 million people were infected, 13,000 patients were hospitalized, and 656 persons died due to the 2009 pandemic H1N1 infection.

It was important to describe the clinical picture and define the risk factors of A(H1N1)pdm09 infection, in order to support public health policy makers in developing vaccination strategies, antiviral use, and other control measures [4]. The clinical and epidemiologic characteristics of the patients hospitalized because of A (H1N1) pdm09 infection were described in the beginning of the outbreak [2,4-7]. However, detailed studies to understand the course of the disease and the predictors of fatality are necessary for a description of such a historical outbreak. Herein, we describe the predictors of fatality among adult hospitalized patients due to A (H1N1) pdm09 infection in Istanbul, Turkey. Description of the clinical features of hospitalized patients in Istanbul, a city with the population around 13 million, will shed light on the obscure areas in fatality and therapy.

## Methods

### Study population

The study was performed by the İstanbul Pandemic influenza study group of The Turkish Society of Clinical Microbiology and Infectious Diseases (KLIMIK). During and after the 2009 Pandemic, all available data of the hospitalized patients in Istanbul were included in the study. The largest 11 hospitals of Istanbul participated in the study. Three of these hospitals were University Hospitals, and eight were training and research hospitals of The Ministry of Health of Turkey. All patients hospitalized with suspected A (H1N1) pdm09 infection who were ≥ 14 years of age were included in the study. In the beginning of the outbreak, all suspected imported cases were hospitalized for the purpose of disease containment regardless of their need for medical support. Accordingly, these imported cases of the first wave of the outbreak were excluded from this study. The patients from the second wave of the outbreak that started in the beginning of September 2009 were hospitalized because of clinical signs and symptoms of the A (H1N1) pdm09 infections.

The laboratory confirmation was performed by the rRT-PCR method provided by the CDC, Atlanta in one of the two National Influenza Reference Laboratories located in the Istanbul Faculty of Medicine, and at the laboratories of one university and one military hospital. Among the hospitalized patients laboratory diagnosis confirmed patients were included in the study. Infectious diseases and clinical microbiology specialists collected data electronically in individual hospitals, and the pooled data were analyzed. The hospital, official administrative and laboratory data were also reviewed for the consistency of the data related to Istanbul. The study was approved by the Medical Ethics Committee of Marmara University Medical Faculty as a non-interventional clinical research with the number of 09.2010.0097.

### Statistical analysis

In univariate analysis, for comparing fatal and survived cases, categorical data were tested by chi square test and t test was used for comparison of the means of two groups (Tables 1 and 2). Parameters found to be statistically significant in univariate analyse, were tested by logistic regression to predict the risk of fatality (Table 3). The independent variables included in the model were early use of neuraminidase inhibitors, nosocomial infection, and having a malignant disease. In analysis STATA (USA, Texas, version 11) was used, with statistical significance set as <0.05.

**Table 1:** Demographic characteristics of the laboratory confirmed A (H1N1) pdm09 infected cases and their risk factors for fatality

	**Fatal n = 22 (%)**	**Survived n = 219 (%)**	**p**
Female gender	10 (45)	143 (65)	0.065
Mean age	37 (sd 17)	35 (sd 16)	0.543
Age ≥ 65	2 (9)	17 (8)	0.826
Morbid Obesity	1 (5)	14 (6)	0.732
Pregnant women	1 (10)	50 (35)	0.105
Comorbid chronic diseases	11 (50)	89 (41)	0.396
Chronic heart disease	3 (14)	24 (11)	0.704
Diabetes mellitus	1 (5)	18 (8)	0.542
Chronic renal disease	2 (9)	11 (5)	0.421
Chronic neurologic disease	2 (9)	11 (5)	0.421
Chronic obstructive lung disease	3 (14)	21 (10)	0.546
Malignancy	3 (14)	5 (2)	0.005
Vaccinated against H1N1	0	3 (0.37)	0.675
Laboratory findings			
Leukocyte count, median	13, 875	8,700	0.046
Thrombocyte count, median	132,000	180,500	0.074
AST, median	59	25	<0.001
ALT, median	65	21	0.004
CPK, median	285	92	0.005
LDH, median	675	248	0.001
C reactive protein, median	31	25	0.135

**Table 2:** Pulmonary findings of the patients

	**Fatal n = 22 (%)**	**Survived n = 219 (%)**	**p**
Abnormal auscultation of the lung	13 (59)	79 (36)	0.034
Bilateral involvement in chest x-ray	18/20 (90)	59/147 (40)	<0.001
Type of involvement in chest x-ray			
Lobar	1/14 (7)	15/137 (11)	0.608
Interstitial	11/17 (65)	52/137 (38)	0.034
Diffuse consolidation	5/16 (31)	20/136 (15)	0.091
Effusion	3/17 (18)	2/134 (1)	<0.001
Need for mechanic ventilation	15 (68)	13 (6)	<0.001

**Table 3:** Univariate and multivariate analyses for the predictors of the fatality

		**Univariate analysis**			**Multivariate analysis**	
	**Odds ratio**	**Confidence interval**	**p**	**Odds ratio**	**Confidence interval**	**P**
Using neuraminidase inhibitors within two days after onset of symptoms	0.33	0.14-0.79	0.13	0.17	0.03-0.77	0.022
Nosocomial infection	10	4.9-23.2	<0.001	5.7	1.84-18	0.013
Presence of malignancy	4.5	1.6-12.4	0.003	3.8	0.66-22.01	0.133

## Results

**Figure 1:**
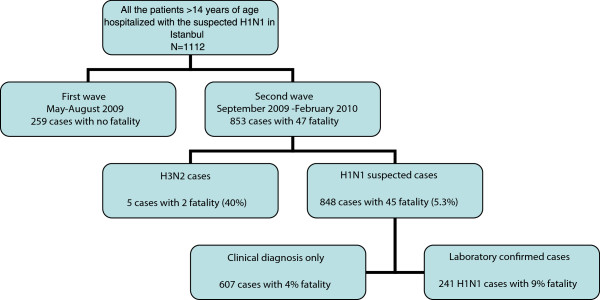
Hospitalized patients with suspected A(H1N1)pdm09 infection in Istanbul between May 2009 and February 2010.

The first cases of Pandemic Influenza A (H1N1) were recorded in May 2009 and the last cases were seen in February 2010. The patients hospitalized during the second wave of the outbreak, which started in September were included in the study (Figure 1). In the second wave of the infection, 848 patients suspected of A (H1N1) pdm09 were hospitalized. Among the hospitalized patients, 9% were admitted to the intensive care unit and 45 (5.3%) died. We limited our analysis with the 241 A(H1N1)pdm09 laboratory confirmed patients (Figure 1). Among 241 laboratory confirmed influenza A(H1N1)pdm09 patients, 22 (9%) died. Nineteen out of 22 fatal cases (86%) were in ICU, whereas 13 out of 219 (6%) survived cases were in ICU (p < 0.001).

All the co-morbid chronic diseases were more common among the fatal cases, but particularly fatality was more commonly observed among patients with malignancies (Table 1). The proportion of pregnant women among female patients was 33%. Out of 51 pregnant women 1 (2%) died, and the fatality rate among pregnant women was less than the non-pregnant women (9%). However, this difference was not statistically significant (p = 0.105). The proportion of the patients with a body mass index > 30, or clinically judged as obese, among fatal and survived cases were found to be similar (p = 0.732). The most common symptoms among H1N1 confirmed patients were fever (89%), cough (87%), shortness of breath (59%), myalgia (56%), headache (54%), sore throat (52%), and diarrhea (10%).

Leukocyte and thrombocyte counts were lower in the fatal group compared to the surviving patients. The median alanine transferase, aspartate transferase, lactate dehydrogenase, creatinin phosphokinase, and C reactive protein levels were higher among fatal cases (Table 1). Pulmonary findings were common among fatal cases (Table 2).

Among laboratory confirmed influenza A(H1N1)pdm09 cases, the median length of stay was 5 days (interquartile range 2 and 9). Secondary nosocomial bacterial infection was detected in 18 out of 241 (7%) patients. The most commonly isolated nosocomial bacterial pathogens were Acinetobacter spp. (n = 4), Methicillin resistant Staphylococcus spp. (n = 4), E.coli (n = 3), Pseudomonas spp. (n = 1), and Klebsiella spp. (n = 1).

Out of 241 laboratory confirmed patients, 222 (93%) received oseltamivir, and 4 (2%) received zanamivir. Among the patients who used neurominidase inhibitors within 48 hours after onset of symptoms the case fatality rate was 2%, whereas among the patients who did not use the CFR it was 13%. Among the hospitalized patients with confirmed diagnosis of H1N1 infection, 69% received at least one antibiotic. In decreasing order, 67 patients (28%) received respiratory quinolones, 64 patients (26%) received macrolides, 35 (15%) third generation cephalosporins, 40 (16%) ampicillin with sulbactam or amoxicillin with clavulanic acid, 25 (10%) carbapenems, 14 (6%) piperacillin-tazobactam. The rate of vaccination against H1N1 was 0.4, whereas against seasonal flu was 7%.

In multivariate logistic regression, early use of neuraminidase inhibitors was found to be protective (Odds ratio: 0.17, confidence interval: 0.03-0.77, p = 0.022), nosocomial infections (OR: 5.7, CI: 1.84-18, p = 0.013), presence of malignant disease (OR: 3.8, CI: 0.66-22.01, p = 0.133) (Table 3).

## Discussion

Istanbul has a population of 13 million according to the 2011 census. Analysis of the pandemic influenza data of Istanbul is valuable because Istanbul has high population and has a potential of being an entrance gate for pandemic infections. Turkey experienced the first wave of the outbreak from May to August, followed by an early 2009 fall influenza season. Usually the influenza season in Turkey starts in December. The majority of the patients in the first wave were imported cases. Since these patients were hospitalized in order to contain the pandemic, their data was not included in this study. The first cases of the second wave were reported between September 2009 and February 2010. Since almost all cases were detected as H1N1 infection in this period, the Ministry of Health of Turkey declared that it was not necessary to confirm the laboratory diagnosis for hospitalized cases. On the other hand, the strongest feature of this study was inclusion of almost all the patients who were hospitalized because of H1N1 in Istanbul. Since the largest state hospitals participated in the study, the goal of presenting a realistic picture of the first pandemic of the 21^st^ century was achieved.

In our study, the mean ages of fatal and survived cases were not statistically different. The fatality rate among the patients over 65 years of age were not found to be higher, although many authors reported that being older than 65 years of age was associated with fatality [8]. According to a systematic analysis which included 44 articles on A (H1N1) pdm09, early in the pandemic the disease occurred overwhelmingly in children and younger adults, and the case fatality rate was 2.9% among confirmed patients [9]. In a study from the USA, which included hospitalized patients, which included also children [10], among 255 hospitalized patients 8% died. In our study, the case fatality rate was 9.1% among the confirmed cases. The majority of the fatalities (75%) occurred within the first 2 weeks of hospitalization. Thirteen percent of confirmed patients were admitted to the intensive care unit. In another multicenter study performed in Turkey, a total of 821 children with 2009 pandemic H1N1 were hospitalized. The majority of admitted children (56.9%) were younger than 5 y of age, and 35 children (4.3%) died. The death rate was significantly higher in patients with malignancy, chronic neurological disease, immunosuppressive therapy, at least 1 pre-existing condition, or respiratory complications [11].

Cough and fever were the most common clinical symptoms as it was reported for the confirmed cases in previous reports [9]. Nosocomial bacterial infection was detected in 7% of the patients. In one study it was reported that bacterial co-infection was common and was associated with age > 50 years and co-morbidities [12]. As one of the different features from seasonal influenza, A (H1N1) pdm09 was reported to pose an increased risk of severe illness in pregnant women [10,13-15]. On the other hand, some studies reported no increased risk of fatality because of A (H1N1) pdm09 infection among the pregnant women [16,17]. In our study group, the proportion of the pregnant women among female patients was 33%, and the case fatality proportion was lower in pregnant women than the non-pregnant women. Because of the awareness about the higher fatality among pregnant women, hospitalization was high. Severely obese individuals with and without chronic conditions were reported to be at increased risk for respiratory hospitalizations during influenza seasons [10,18-20]. However, in our study the proportion of obese patients with body mass index of >30 or clinically judged, among fatal and survived cases were found to be similar (p = 0.912, Table 1). Lack of association of obesity and pregnancy with fatality could be related to low power of the study.

Early use of oseltamivir was reported to be beneficial in treatment [8,10,21,22]. Furthermore, it was reported that among the patients with A(H1N1)pdm09 infection, early use of antiviral therapy prevented development of pneumonia [23,24]. We found that the patients who received neuraminidase inhibitors within 2 days after the disease onset were found to be less likely to die in comparison with the patients who received neuraminidase inhibitors later than two days (Odds ratio: 0.16, confidence interval: 0.03-0.74, p = 0.019, Table 3). According to a recent study from USA, chest radiographs obtained at hospital admission revealed pneumonia in 103 (46%) of 255 patients. Among 255 hospitalized patients, 208 (82%) received neuraminidase inhibitors, but only 47% had treatment ≤ 2 days after illness onset [10]. The rate of vaccination against A (H1N1) pdm09 was 0.4 and was much lower than the rate of vaccination against seasonal flu. Unnecessary use of antibiotics was common among patients who were hospitalized for A (H1N1) pdm09. One of the reasons for such a high rate of unnecessary antibiotic use was obtaining the A (H1N1) pdm09 test results requires an average of 2–3 days. Early diagnosis of influenza virus infection will decrease unnecessary use of antibiotics.

## Conclusion

In Influenza A (H1N1) pdm09 infection, using neuraminidase inhibitors within 2 days after onset of symptoms decreased, whereas nosocomial bacterial infections and underlying malignant diseases increased CFR.

## Competing interest

The authors declare that they have no competing interests. This study was performed by the Pandemic Influenza Study Group of Turkish Society of Clinical Microbiology and Infectious Diseases.

## Authors’ contributions

Substantial contributions to conception and design, or acquisition of data, or analysis and interpretation of data. EÖ, AS, AÖ, SF, KA, GA, ED, ÖO, Bİİ, CB. involved in drafting the manuscript or revising it critically for important intellectual content. Ergönül ÖBN, YS ŞF, UN, İA, EG, CMA, MK, OM. Final approval of the version to be published. BS, GS, NaÖ, ÖS, ÖN, YT, AT, GP, SN, FM, DAİ, EH. All authors read and approve the final manuscript.

## Pre-publication history

The pre-publication history for this paper can be accessed here:

http://www.biomedcentral.com/1471-2334/14/317/prepub
